# Melanin-concentrating hormone attenuates the hedonic feeding induced by orexin-A in the ventral tegmental area of high-fat diet male mice

**DOI:** 10.3389/fnut.2024.1468874

**Published:** 2024-12-20

**Authors:** Xiaoning Liu, Helin Yang, Wenguang Xu, Xuezhe Wang, Wenhui Tang, Xiaoxuan Wang, Yang Jiao, Xinchi Luan, Pengmeng Li, Feifei Guo

**Affiliations:** ^1^Department of Pathophysiology, School of Basic Medicine, Qingdao University, Qingdao, Shandong, China; ^2^Department of Pathology, Women and Children’s Hospital, Qingdao University, Qingdao, Shandong, China; ^3^Department of Spine Surgery, Peking University People’s Hospital, Women and Children’s Hospital, Qingdao University, Qingdao, Shandong, China; ^4^Department of Gastroenterology, Affiliated Qingdao Third People’s Hospital, Qingdao University, Qingdao, Shandong, China

**Keywords:** lateral hypothalamic area, ventral tegmental area, melanin-concentrating hormone, orexin-A, hedonic feeding

## Abstract

**Objective:**

The ventral tegmental area (VTA), a pivotal hub in the brain’s reward circuitry, receives inputs from the lateral hypothalamic area (LHA). However, it remains unclear whether melanin-concentrating hormone (MCH) and orexin-A (OX-A) neurons in the LHA exert individual or cooperative influence on palatable food consumption in the VTA. This study aims to investigate the modulatory role of MCH and OX-A in hedonic feeding within the VTA of high-fat diet (HFD) mice.

**Methods:**

Male mice were subjected to an 8-week high-fat diet. To visualize the projections from the LHA to VTA, we employed fluorescent gold retrograde tracing combined with immunofluorescence staining. Immunofluorescence staining or enzyme-linked immunosorbent assay was used to detect the activity of the VTA neurons, expression of OX-A or MCH in the LHA, as well as the activity of their receptors (OXR1 and MCHR1) in the VTA following a sucrose preference test. Single-unit extracellular electrical discharge recordings were conducted to assess the effects of OX-A and MCH on VTA neurons in HFD mice. Additionally, chemogenetic inhibition of MCH neurons and immunofluorescence staining were utilized to observe the regulatory roles of MCH in changes of hedonic feeding induced by OX-A in HFD mice.

**Results:**

Sucrose intake resulted in lower activation of VTA neurons in the HFD mice compared to CON mice, while OX-Aergic and MCHergic neurons project from the LHA to the VTA. Although sucrose intake increased the expression of OX-A and MCH in HFD mice, it led to diminished activation of OXR1-positive and MCHR1-positive VTA neurons compared to CON mice. Extracellular single-unit recording revealed that MCH significantly suppressed the firing rate of OX-A-responsive neurons in the VTA. MCH attenuated the hedonic feeding response induced by OX-A in HFD mice, and administration of MCHR1 antagonist (SNAP94847) significantly potentiated the effect of OX-A. Chemogenetic inhibition of MCH neurons improved the activity of OXR1-expressing neurons, which could be reversed by pretreatment with an OXR1 antagonist (SB334867). Furthermore, chemogenetic inhibition of MCH enhanced hedonic feeding behavior, which was counteracted by SB334867 treatment in HFD mice.

**Conclusion:**

Melanin-concentrating hormone could attenuate the hedonic feeding behavior induced by orexin-A in the VTA of HFD mice.

## Introduction

Obesity is a well-established and escalating global health issue, elevating the risk of mortality, type 2 diabetes, cardiovascular disease, and compromising overall quality of life (1). One significant contributing factor to obesity is compulsive-like overconsumption of highly palatable and high-fat foods, which usually induces “pleasure” to drive hedonic feeding (2, 3). It is known that mostly hedonic feeding is not satisfying the physiological requirement, and always exhibits behavioral and substance addiction characteristics (4). Preclinical studies have demonstrated that male rats with high-fat diet (HFD)-induced obesity exhibit similar alterations in neural circuitry as observed in animal models of substance abuse (5, 6).

Ventral tegmental area (VTA) is a pivotal midbrain region that plays a fundamental role in reward and motivation. Over 70% of VTA neurons are dopaminergic, and the projection from the VTA*^DA^* neurons to the nucleus accumbens (NAc) primarily encodes reward-associated memory and reinforcement learning (7). Simultaneously, the VTA*^DA^* neurons receive both excitatory and inhibitory inputs from a broad distribution of brain areas, including the lateral hypothalamus area (LHA), the lateral orbitofrontal cortex, the ventral striatum and dorsal striatum (8).

The LHA coordinates a repertoire of fundamental behaviors, encompassing feeding, sleep-wake cycles, stress and motivated behavior (9). These functions rely on the neural afferent/efferent interactions of LHA and neurotransmitters (10). Both orexin-A (OX-A) and melanin-concentrating hormone (MCH) are neuropeptides primarily expressed in the LHA. OX-A modulates hyperarousal states, reward processing, and addiction by influencing downstream nuclei including locus coeruleus, VTA, basal forebrain to paraventricular thalamus, among others (11). The affinity of OX-A for the orexin receptor OXR1 is ten times higher than OXR2, and only OXR1 is expressed in the medial prefrontal cortex and the hippocampus. On the other hand, OXR2 is also expressed in hypothalamic regions such as the arcuate nucleus and paraventricular nucleus. Both OXR1 and OXR2 are expressed in the amygdala, bed nucleus of the stria terminalis, and VTA (12). Previous studies have demonstrated that OX-A administration leads to an increase in firing frequency and burst firing of majority of VTA*^DA^* neurons (13). Borgland et al., further proved that OX-A selectively enhances firing of the VTA*^DA^* neurons projecting to the lateral shell of the NAc (lAcbSh) and the medial shell of the NAc (AcbSh) (14). Functionally, calorie restriction-induced release of OX-A has been shown to augment glutamate synaptic strength on VTA*^DA^* neurons and enhance motivation for food-seeking behavior (15). These findings suggest a potential role for OX-A in modulating feeding motivation through VTA*^DA^* neurons. However, its impact on hedonic feeding via VTA*^DA^* neurons in obesity remains unclear.

Melanin-concentrating hormone, an amino acid cyclic peptide, is primarily distributed in the LHA and zona incerta (ZI) (16). Its main functions encompass energy homeostasis, reward processing, sleep regulation, learning and memory processes, as well as social interactions (17). MCH exerts its effects through interaction with receptors MCHR1 and MCHR2. Notably, in rodents, the presence or functional activity of MCHR2 is either absent or minimal; conversely, MCHR1 exhibits widespread expression throughout the brain (12). Although Tatiana et al., demonstrated that MCH did not influence VTA neuron firing in coronal brain slices from 3- to 4-week-old male Wistar rats (13), a significant reduction in Fos-immunopositive VTA*^DA^* neurons was observed in female MCHR1 KO mice (18), suggesting the involvement of MCH in modulating reward behaviors. Furthermore, it has been reported that MCH may inhibit DAergic cells within the VTA while disinhibiting local glutamatergic signaling to restore DA levels (19).

OX-A and MCH originate from distinct neuronal populations within the LHA, yet both exhibit widespread projections to overlapping brain regions. The available evidence indicates that OX-A and MCH play complementary roles in regulating the feeding behavior, potentially through exerting contrasting effects on reward systems, motivation, as well as various physiological processes including the sleep-wake cycle, energy usage, and glucose metabolism (20). Despite acknowledging their involvement in promoting food intake, it remains unclear how OX-A and MCH individually or cooperatively modulate consumption of palatable foods. Further investigation is imperative to elucidate the intricate interplay between OX and MCH in regulating hedonic feeding within the VTA.

In this study, we hypothesized that the motivation for food seeking driven by OX-A is modified by HFD, while MCH regulates the excitatory effect of OX-A on the VTA*^DA^* neurons. We employed Fluoro-Gold (FG) retrograde tracing combined with immunofluorescence staining to demonstrate the projections of OX-Aergic and MCHergic neurons from the LHA to VTA. Additionally, we assessed VTA neuron activity, expression levels of OX-A and MCH in the LHA, as well as the activation of their receptors (OXR1 and MCHR1) in the VTA following a sucrose preference test. Furthermore, we investigated the effects of exogenous OX-A and MCH on VTA neurons in both control and HFD mice. To evaluate hedonic behavior changes induced by OX-A in HFD mice, chemogenetic technology and immunofluorescence staining were utilized to observe the regulatory roles of MCH in VTA. Overall, our study aims to investigate the modulatory role of the MCH and OX-A in the hedonic feeding within the VTA region specifically in HFD mice, potentially providing a therapeutic target for managing hedonic behavior associated with obesity.

## Materials and methods

### Animals and high-fat diet

Healthy male KunMing mice (3-week-old) were utilized in this study (Animal protocol number: SCXK (Lu) 20190003). They were housed under controlled conditions at a temperature of 22 ± 2°C with a 12-h light/dark cycle (lights on 08:00 - 20:00) and provided *ad libitum* access to food and water. Following an adaptation period, 4-week-old mice were randomly assigned to receive an 8-week high-fat diet (Research Diets D12492, Beijing Keao Xieli Feed Co., Ltd.) – from which 60% of calories were derived from fat, 20% from protein and 20% from carbohydrates, resulting in the development of HFD mice. The control mice were fed D12450B (10 kcal% fat diet). All experimental procedures were conducted following approval from the Qingdao University Animal Care and Use Committee.

### Experimental design

Experiment 1: Mice were assigned to control (CON) and HFD groups (*n* = 9) by simple randomization, the c-Fos expression in the VTA, OX-A and MCH expression in the LHA, activated OXR1 or activated MCHR1 in the VTA were observed respectively 30 min after sucrose preference testing.

Experiment 2: 1 week after injection of FG in the VTA, mice were randomly selected for retrograde tracking combined with immunofluorescent staining to observe the coexistence of FG and MCH/OX-A immunoreactive neurons in the LHA (*n* = 6).

Experiment 3: Twenty-five CON mice and 18 HFD mice were randomly selected for single-unit extracellular electric discharge recording.

Experiment 4: Thirty-six CON mice were assigned to four groups by simple randomization. The VTA of CON mice were microinjected normal saline (NS), OX-A, MCH, or MCH + OX-A seperately. Thirty-six HFD mice were assigned to four groups by simple randomization. The VTA of HFD mice were microinjected NS, OX-A, MCHR1 antagonist SNAP94847 (SNAP), or SNAP + OX-A (*n* = 9). Sucrose preference testing was conducted following a 7-day-administration in the VTA.

Experiment 5: HFD mice were assigned to four groups: NS + NS, SNAP + NS, NS + clozapine N-oxide (CNO), and SNAP + CNO by simple randomization (*n* = 9). These four groups were given intraperitoneal administration of NS or CNO and VTA microinjection of NS or SNAP. Adeno-associated virus (AAV) vector (AAV2/2-rMCHp-hM4D(Gi)-mCherry-WPRE-pA) was stereotaxically injected into the LHA of the mice. After 2 weeks of recovery, cannula implantation was performed in the VTA. One week later, OXR1 antagonist SB334867 (SB) was microinjected into the VTA for seven consecutive days. On the day of the experiment, CNO was dissolved in saline and intravenously administered 30 min prior to the behavior tests to inhibit MCHergic neurons in the LHA. Following sucrose preference testing, immunoreactive MCH and mCherry in the LHA, MCH expression in the LHA, OXR1 and c-Fos expression in the VTA were observed respectively.

### Retrograde tracing and immunohistochemistry

In order to visualize the projections from LHA to VTA, the HFD mice were randomly selected and subjected to overnight fasting. The mice were anesthetized using 1.4–1.5% isoflurane in combination with oxygen (at a flow rate of 1.0 L/min) after thiobutabarbital (100 mg/Kg, i.p.) pretreatment. They were then immobilized on stereotaxic apparatus (Narashige SN-3, Tokyo, Japan). Following coordinates provided by Paxinos and Franklin’s mouse brain atlas (2001), 100 nL of 3% Fluoro-Gold (FG, Sigma-Aldrich Chemical, MO, USA) was injected into VTA (bregma: P: −2.92 mm, L: 0.6 mm, H: 4.3 mm). After a 7-day period, the mice were anesthetized again and transcardially perfused with saline solution, followed by fixation with 4% paraformaldehyde in phosphate buffer solution. The brains were immediately removed and post-fixed in 4% paraformaldehyde for 2 h before being transferred to a sucrose solution (30%) at a temperature of 4°C for at least 24 h. Subsequently, serial frontal sections measuring approximately 15 μm thick were obtained using a freezing microtome (Kryostat 1720, Leica, Germany). The brain sections were incubated overnight at 4°C with anti-orexin-A (rabbit, 1:500, ab255294, Abcam)/anti-MCH (rabbit, 1:500, ab274415, Abcam)/anti-c-Fos (mouse, 1:800, ab208942, Abcam) antibodies, followed by incubation with goat anti-rabbit Cy3 antibodies (1:300, ab6939, Abcam)/goat anti-mouse Cy3 antibodies (1:500, ab97035, Abcam) for 2 h at room temperature. A fluorescence microscope (DM6000B, Leica Microsystems AG, Wetzler, Germany) was utilized to observe FG and immuopositive neurons in the LHA. Five fields of five brain slices from each mouse were analyzed for immunoreactive cells within an area of 350 × 350 μm^2^. The percentage of double-labeled cells was calculated as follows: (%) = the numbers of double-labeled cells/the numbers of MCHR1/OXR1 positive neurons × 100%.

### Implantation of brain cannulae and drug administration

The HFD mice were subjected to a 12-h fasting period prior to anesthesia with isoflurane, followed by their placement in the stereotactic apparatus. To administer the drug, a guide cannula (RWD Life Science Co., Ltd.) was implanted into VTA (bregma: P: −2.92 mm, L(R): 0.6 mm, H: 4.3 mm) and secured with dental acrylic cement. After surgery, a 7-day recovery period was allowed for the mice. Subsequently, 1.5 μL of OX-A (10.0 μg/μL, dissolved in NS, Sigma, St. Louis, MO, USA), MCH (1.0 μg/μL, dissolved in NS, Sigma, St. Louis, MO, USA), SNAP (2.0 μg/μL, dissolved in NS, Sigma, St Louis, MO, USA), SB (40.0 μg/μL, dissolved in NS, St Louis, MO, USA) or NS were administered to the mice using an injection cannula connected to a syringe via a polyethylene tube (21, 22).

### Extracellular single-unit recording

For investigating the effects of drugs on the activity of DA neurons in the VTA, the single-unit extracellular recordings *in vivo* were performed as previously described (23, 24). The mice were anesthetized by isoflurane and secured in the stereotaxic apparatus. A five-tube glass microelectrode (tip diameter: 3–10 μm, resistance: 5–15 M) were stereotactically lowered into the VTA (Bregma: P: −2.92 mm, L: ± 0.6 mm, H: 4.3 mm) according to the Paxinos and Franklin’s brain atlas (2001) to collect single-unit discharge recordings and to perform micropressure injections. The recording barrel of the electrode was filled with 0.5 M sodium acetate and 2% pontamine sky blue. The other four barrels were filled with OX-A (15 nM, Sigma, St Louis, MO, USA), MCH (200 nM, Sigma, St Louis, MO, USA), OX-A + MCH (15 and 200 nM) or saline for the CON mice and with OX-A (15 nM), SNAP (25 nM, Sigma, St Louis, MO, USA), OX-A + SNAP (15 and 25 nM) or saline for the HFD mice. The drugs were infused on the surface of neurons using a short pulse of gas pressure (1500 ms, 5.0 ∼ 15.0 psi) from a pressure injector (PM2000B; Micro Data Instrument Inc., NJ, USA), and the injection volume of each drug was less than 1 nL. The doses of drugs were selected based on those reported in previous studies (25, 26). When the glass microelectrode was lowered into the VTA, action potentials were recorded from a single cell at a time. Once the firing pattern had been stable for at least 120 s, spontaneous firing of VTA*^DA^* neurons were identified by the location, extracellular waveform, firing rate and pattern (27, 28). OX-A was ejected on the surface of DA neuron from one barrel of the microelectrode. If the mean firing rate increased or decreased at least 20% from the mean basal level, the DA neuron was named OX-excited (OX-E) neuron. No respond to OX-A or MCH was OX-N or MCH-N. Subsequently, approximately 5 min after the neuronal firing stabilized, MCH was released on the same OX-E neuron. If the mean firing rate of this neuron changed ≥20% from the mean basal level, the cell was identified as an OX-E & MCH-E/MCH-I neuron. In the CON mice, the mixture of OX-A and MCH was administrated to research the regulation of MCH on the effect of OX-A on the VTA*^DA^* neurons. In the HFD mice, the effects of MCHR1 antagonist SNAP on the DA neurons were studied. To visualize the extracellular action potentials on an oscilloscope (VC-II, Nihon Kohden, Tokyo, Japan), they were recorded by a MEZ8201 amplifier (Nihon Kohden, Tokyo, Japan) and imported into a SUMP-PC biological signal processing system. All data were resaved for analysis.

### Sucrose preference testing

Sucrose preference testing was conducted over a 3-day period, consisting of two habituation days followed by one experiment day. During the initial habituation days, mice were provided with two water bottles, one containing 1% sucrose and the other containing tap water. On the subsequent habituation day, the positions of 1% sucrose and water bottles were reversed to eliminate any potential bias toward a particular side. On the experiment day, both bottles were removed for a 4-h liquid deprivation period. After this period, mice were given access to 1% sucrose and water separately for 30 min each. Following another 4-h interval, each mouse was presented with two separate bottles containing either 1% sucrose or water. The consumed volume of each liquid was recorded and used to calculate the sucrose preference index using [sucrose solution consumed (ml) - water consumed (ml)]/[sucrose solution consumed (ml) + water consumed (ml)] (21).

### Enzyme-linked immunosorbent assay (ELISA)

The procedure was performed as previously described (21). After the sucrose preference testing, the HFD and control group mice were anesthetized. The brains of mice were carefully extracted, ensuring preservation of the LHA within coordinates ranging from −0.58 to −1.58 mm, in accordance with the stereotaxic mouse brain atlas by Paxinos and Franklin (29). Subsequently, the isolated tissues were promptly flash frozen using dry ice and stored at −80? until further analysis. To determine the concentrations of OX and MCH in the LHA, commercially available OX-A ELISA test kits recommended by Jianglaibio (JL23317) and MCH ELISA test kits recommended by Jingmei Biotechnology (JM-11402M1) were employed following manufacturer instructions.

### Chemogenetic technology

The mice received a stereotaxic injection of a recombinant adeno-associated virus (AAV) vector (AAV2/2-rMCHp-hM4D(Gi)-mCherry-WPRE-pA, titer: 5E + 12 vg/mL, volume: 0.2 μL) targeting the LHA region (P: 1.34 mm, L: ± 1.2 mm, and D: 5.0 mm). A volume of 0.5 μL AAV was bilaterally injected using the syringe at a controlled rate of 0.1 μL/min, followed by its removal after a duration of 10 min post-injection for optimal delivery efficiency. Three weeks following the AAV injection into LHA, intraperitoneal administration of CNO dissolved in NS (1 mg/mL, 0.15 mg/kg) effectively inhibited MCHergic neurons within this region. Then the sucrose preference testing were conducted to evaluate the hedonic behaviors (21).

### Histological verification

To confirm the recording position of the glass micro-electrode, a spot of pontamine sky blue was generated at the recording site by applying a direct current (10 μA, 20 min) through the electrode. Subsequently, perfusion and fixation of each mouse brain was performed to verify the accuracy of recording and injection sites. Frozen coronal sections with a thickness of 50 μm were then obtained from either the VTA or LHA for further analysis. Incorrectly positioned data or unilateral expression in the chemogenetic experiments was excluded from statistical analysis.

### Statistics

The smallest sample size needed for the experiments is calculated by a power analysis. The data were analyzed with Prism 6 software (GraphPad Software, San Diego, CA, United States) and presented as mean ± standard deviation. Student’s *t*-tests were employed to compare differences between two groups. A significance level of *P* < 0.05 was considered statistically significant.

## Results

### Reduced VTA neuronal activity of the HFD mice in response to sucrose intake

**FIGURE 1:**
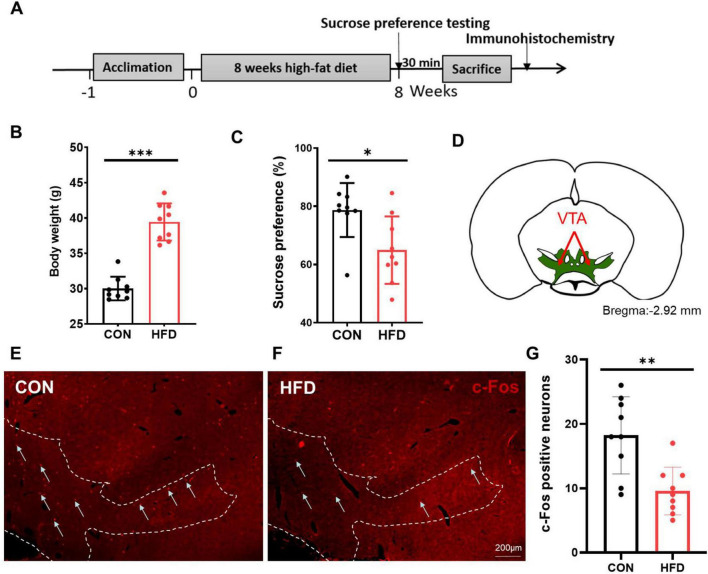
HFD mice had reduced activity in the VTA to sucrose intake. **(A)** The timeline of Experiment 1. **(B)** The body weight of the CON and HFD mice. **(C)** The sucrose preference index of the CON and HFD mice. **(D)** The schematic diagram of the VTA mapped on coronal atlas (shown as green area). **(E)** C-Fos expression in the VTA of the CON mice after sucrose intake. **(F)** C-Fos expression in the VTA of the HFD mice after sucrose intake. **(G)** The number of c-Fos positive cells in the VTA between the CON and the HFD mice. Scale bars, 200 μm. VTA, the ventral tegmental area. *P < 0.05, **P < 0.01, ***P < 0.001, *n* = 9.

After 8 weeks (Figure 1A), the body weight of the HFD group exhibited a significant increase compared to that of the CON group (*P* < 0.001, *t* = 9.068, *df* = 16, Figure 1B). In the sucrose preference test, the HFD mice displayed reduced sucrose consumption compared to the CON mice (*P* < 0.05, *t* = 2.786, *df* = 16, Figure 1C). To characterize alterations in the reward system among HFD mice, we assessed the change of c-Fos expression after the sucrose preference testing in the VTA (Figure 1D). Figures 1E, F demonstrate a significantly lower number of c-Fos positive cells in the VTA within the HFD group (9.56 ± 3.71) compared to the CON group (18.22 ± 5.99, *P* < 0.01, *t* = 3.687, *df* = 16, Figure 1G).

### Projections of OX-A and MCH neurons from the LHA to the VTA

**FIGURE 2:**
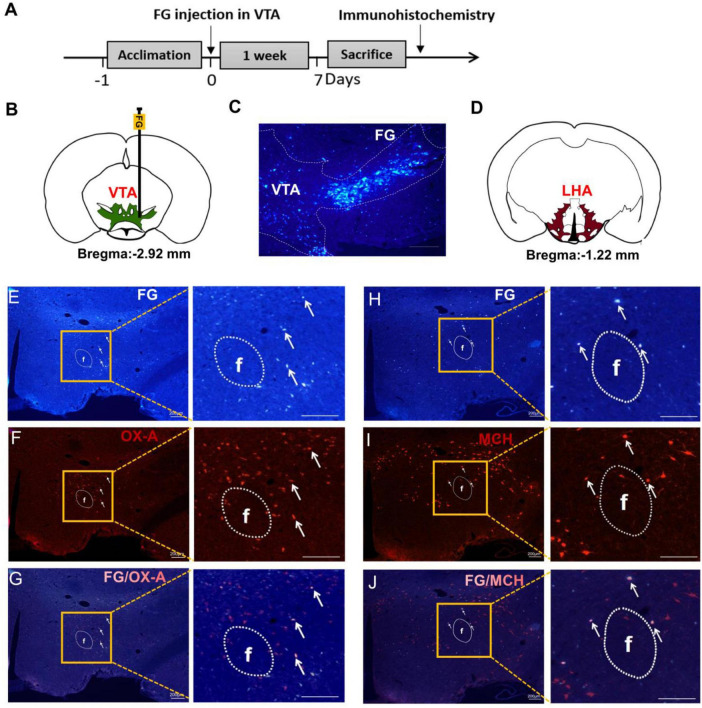
OX-A or MCH immunopositive neurons were co-labeled by FG retrograding from the VTA. **(A)** The timeline of Experiment 2. **(B)** A schematic diagram of FG injection in the VTA. **(C)** A representative image of FG injected into the VTA. **(D)** The location of the LHA mapped on coronal atlas (shown as brown area). **(E)** The retrograde tracing of FG from VTA to LHA. White arrows indicate FG-labeled neurons. **(F)** The expression of OX-A in the LHA. White arrows indicate OX-A immunopositive neurons. **(G)** The merge of OX-A and FG in the LHA. White arrows indicate co-labeling of OX-A and FG. **(H)** The retrograde tracing of FG from VTA to LHA. White arrows indicate FG-labeled neurons. **(I)** The expression of MCH in the LHA. White arrows indicate MCH immunopositive neurons. **(J)** The merge of MCH and FG in the LHA. White arrows indicate co-labeling of MCH and FG. Scale bars, 200 μm. *n* = 6. LHA, the lateral hypothalamic area; VTA, the ventral tegmental area; FG, fluorescent gold; f, fornix.

In order to investigate the afferent projections modulating the VTA neurons, retrograde tracing was performed by injecting FG into the VTA (Figures 2A–C). Seven days later, FG was observed in the LHA (Figure 2D). Figures 2E, H showed that there were some FG labeled LHA neurons. Combined with the OX-A and MCH immunofluorescence staining (Figures 2F, I), the co-labeled neurons by FG and OX-A or FG and MCH were found in the LHA (Figures 2G, J). Cell counting showed that 17.82 ± 3.18% of LHA neurons were co-labeled with both FG and OX-A, and 20.15 ± 3.97% were co-labeled with both FG and MCH.

### Increased expression of OX-A and MCH in the HFD mice after sucrose intake

**FIGURE 3:**
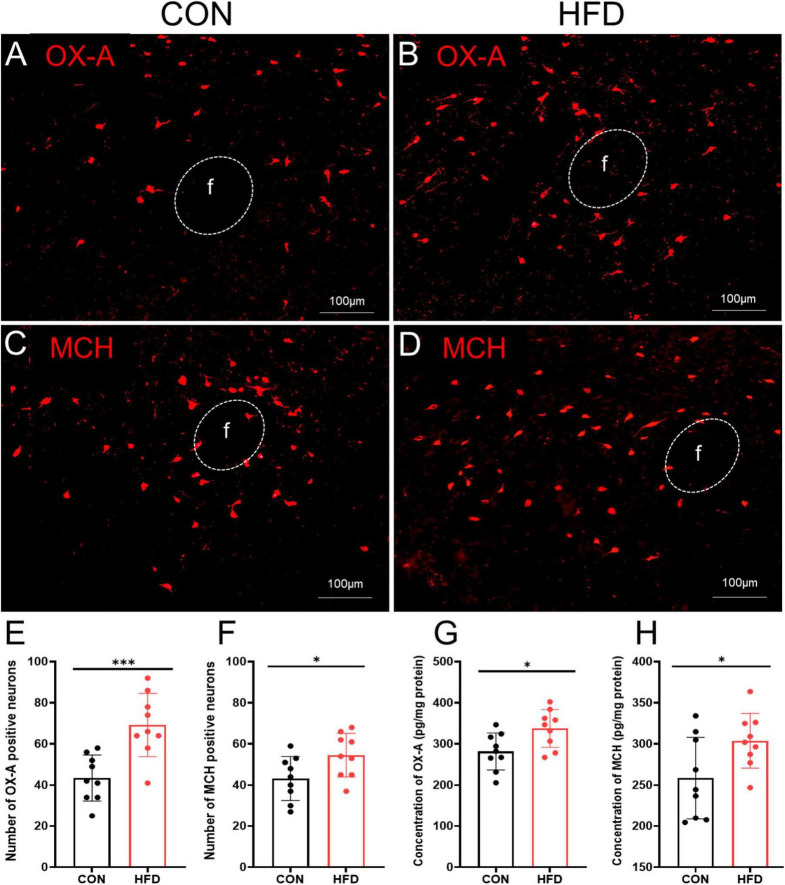
OX-A and MCH were highly expressed in the HFD mice after sucrose intake. **(A)** The expressions of OX-A in the CON mice. **(B)** The expressions of OX-A in the HFD mice. **(C)** The expressions of MCH in the CON mice. **(D)** The expressions of MCH in the HFD mice. **(E)** The number of OX-A immunopositive neurons in the CON mice and HFD mice. **(F)** The number of MCH immunopositive neurons in the CON mice and HFD mice. **(G)** The concentration of OX-A of the LHA in the CON mice and HFD mice. **(H)** The concentration of MCH of the LHA in the CON mice and HFD mice. Scale bars, 100 μm. f, fornix. *P < 0.05, ***P < 0.001, *n* = 9.

The expression of OX-A and MCH in the LHA was investigated following sucrose intake in CON and HFD mice. The number of OX-A immunopositive neurons was significantly higher in the HFD mice (69.22 ± 15.36) compared to the CON mice (43.44 ± 11.18, *P* < 0.001, *t* = 4.070, *df* = 16, Figures 3A, B, E), and ELISA results also showed that the concentration of OX-A in the hypothalamus was obviously increased in the HFD group (337.62 ± 45.93 pg/mg protein) compared to the CON group (281.77 ± 44.99 pg/mg protein, *P* < 0.05, *t* = 2.606, Figure 3G). Similarly, there was more MCH immunopositive neurons (54.56 ± 10.62) in the HFD group than CON group (43.22 ± 10.70, *P* < 0.05, *t* = 2.256, *df* = 16, Figures 3C, D, F) and higher MCH concentration in the HFD group (303.81 ± 33.21 pg/mg protein) compared to the CON group (258.44 ± 49.64 pg/mg protein, *P* < 0.05, *t* = 2.279, *df* = 16, Figure 3H).

**FIGURE 4:**
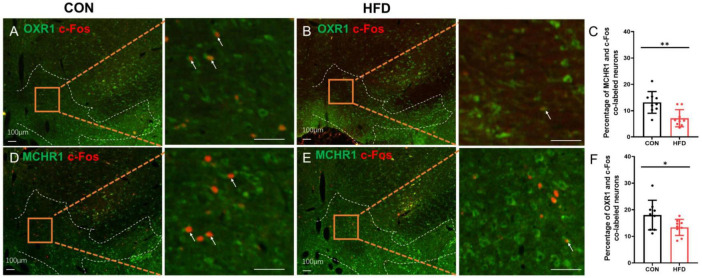
The OXR1 and MCHR1 expressing neurons in the VTA had a lower activity after sucrose intake. **(A)** The co-expressions of OXR1 and c-Fos in the CON mice. **(B)** The co-expressions of OXR1 and c-Fos in the HFD mice. **(C)** The percentage of OXR1 and c-Fos co-labeled cells in the CON mice and HFD mice. **(D)** The co-expressions of MCHR1 and c-Fos in the CON mice. **(E)** The co-expressions of MCHR1 and c-Fos in the HFD mice. **(F)** The percentage of MCHR1 and c-Fos co-labeled cells in the CON mice and HFD mice. Scale bars, 100 μm. *P < 0.05, **P < 0.01, *n* = 9.

Additionally, c-Fos expression was employed to demonstrate the neuronal activity of OXR1 or MCHR1 expressing neurons in the VTA. Interestingly, following sucrose consumption, the HFD mice exhibited a decreased proportion of co-labeled OXR1/c-Fos (13.38 ± 3.02%) in the VTA compared to the CON mice (18.00 ± 5.56%, *P* < 0.05, *t* = 2.191, *df* = 16, Figures 4A–C). Additionally, there was a reduced proportion of co-labeled MCH/c-Fos (7.17 ± 3.24%) in HFD mice compared to CON group (13.17 ± 4.12%, *P* < 0.01, *t* = 3.433, *df* = 16, Figures 4D–F).

### Different responses of the VTA neurons induced by OX-A and MCH in the CON and HFD mice

**FIGURE 5:**
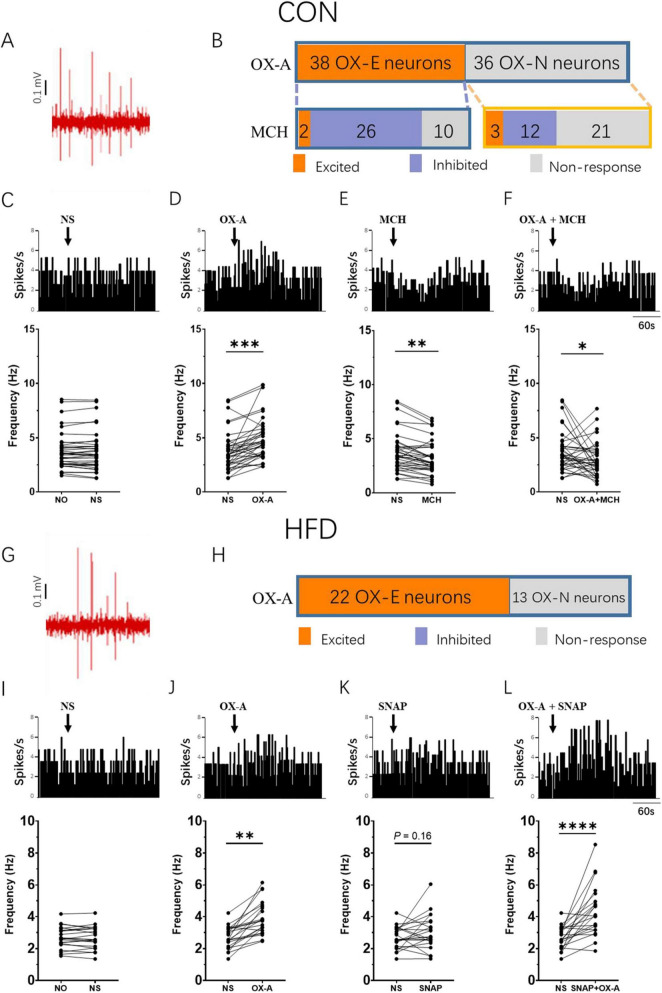
MCH decreased the firing rate of OX-E neurons in the VTA. **(A)** A representative image of the position where the spontaneous firing of VTA neurons was recorded via glass microelectrode (blue point). **(B)** Example of CON mice VTA DA neurons spontaneous firing *in vivo*. **(C)** The number of OX-A and MCH responsive neurons in CON mice. **(D–G)** The representative changes of OX-E neurons firing rate modulated by NS, OX-A, MCH, and OX-A + MCH in the CON mice. **(H)** Example of HFD mice VTA DA neurons spontaneous firing *in vivo*. **(I)** The number of OX-A responsive neurons in HFD mice. **(J–M)** The representative changes of OX-E neurons firing rate modulated by NS, OX-A, SNAP, and OX-A + SNAP in the HFD mice. Scale bars, 500 μm. OX-E, the OX-A excited neuron; VTA, the ventral tegmental area; NO, no stimulation; NS, normal saline; SNAP, SNAP94847. *P < 0.05, **P < 0.01, ***P < 0.001, ****P < 0.0001 vs NS group. *n*_*CON*_ = 25, *n*_*HFD*_ = 18.

To investigate the influence of OX-A and MCH on the VTA neurons in the CON mice, the firing rate of VTA neurons was recorded in experiment 3. A total of 74 neurons were recorded in the VTA. The representative position of microelectrode was shown in Figures 5A, B displayed the typical spikes of VTA*^DA^* neurons spontaneous firing. 38 neurons were activated by OX-A with a significant increasing of firing rate from 3.74 ± 1.79 Hz to 4.81 ± 1.95 Hz (OX-E, *P* < 0.001 vs NS treatment, *t* = 5.091, *df* = 37, Figures 5C–E), and the rest did not respond to OX-A (OX-N, 3.53 ± 2.12 Hz vs 3. 73 ± 1.98 Hz, *P* > 0.05 vs NS treatment, Figure 5C). Further, 38 OX-E neurons, treated with MCH, were divided into MCH-excited (OX-E & MCH-E, 2 neurons), MCH-inhibited (OX-E & MCH-I, 26 neurons), and MCH-non-responsive (OX-E & MCH-N, 10 neurons) groups (Figure 5C). The average firing rate of 38 OX-E neurons was decreased by MCH to 3.13 ± 1.56 Hz (*P* < 0.01, *t* = 4.451, *df* = 37, Figure 5F). The mixture of OX-A and MCH inhibited the firing rate of OX-E cells (2.88 ± 1.57 Hz, *P* < 0.05, *t* = 2.160, *df* = 37, Figures 5B, E). For OX-N neurons, MCH activated three neurons (OX-N & MCH-E), inhibited 12 (OX-N & MCH-I), and did not affected 78 (OX-N & MCH-N, Figure 5C).

In the HFD mice, the spontaneous firing rate of VTA*^DA^* neurons was 2.69 ± 0.69 Hz (Figure 5H). A total of 35 neurons were recorded in the VTA, out of which 22 neurons were identified as OX-E neurons and showed significant activation by OX-A, resulting in an increase in firing rate from 2.69 ± 0.69 Hz to 3.92 ± 1.05 (*P* < 0.01, *t* = 6.088, *df* = 21, Figures 5I–K). The remaining thirteen neurons belonged to the OX-N neurons. Due to the upregulation of MCH expression observed in HFD mice, there was just a trend toward increased firing rate following administration of the MCHR1 antagonist SNAP (2.69 ± 0.69 Hz vs 3.01 ± 1.09 Hz, *P* = 0.16, *t* = 1.475, *df* = 21, Figure 5L). Notably, co-administration of OX-A and SNAP resulted in a further significant elevation of firing rate (4.36 ± 1.59 Hz, *P* < 0.0001, *t* = 4.870, *df* = 21, Figure 5M).

### Effects of MCHR1 antagonist SNAP on the change of hedonic feeding induced by OX-A in the HFD mice

**FIGURE 6:**
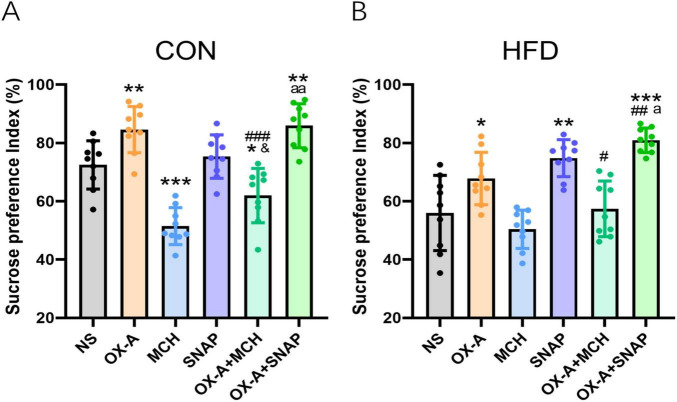
MCH inhibited the change of hedonic behaviors induced by OX-A in the CON and HFD mice. **(A)** The effect of OX-A and MCH on the sucrose preference index (%) of the CON mice. OX-A injection in the VTA increased the sucrose preference index, and MCH injection inhibited the index with or without OX-A treatment. **(B)** The effect of OX-A and MCH on the sucrose preference index (%) of the HFD mice. OX-A treatment increased the index, and the administration of MCH decreased the effect of OX-A on the index. The MCHR1 antagonist SNAP increased the index with or without OX-A treatment. *P < 0.05, **P < 0.01, ***P < 0.001 vs NS group. ^#^*P* < 0.05, ^##^*P* < 0.01, ^###^*P* < 0.001 vs OX-A group. ^&^*P* < 0.05 vs MCH group. *^a^P* < 0.05, *^aa^P* < 0.01 vs SNAP group. *n* = 9.

Based on the electrophysiology results, the sucrose preference test was further used to study the modulatory effect of MCH on the change of hedonic behaviors induced by OX-A in the experiment 4. In the CON mice (Figure 6A), OX-A alone microinjection to the VTA significantly increased the sucrose preference index (84.58 ± 7.93%) compared to CON group (72.47 ± 8.28%, *P* < 0.01, *t* = 3.168, *df* = 16). The index of the MCH group is significantly lower than that of the NS group (51.49 ± 6.33%, *P* < 0.001 vs NS group, *t* = 6.039, *df* = 16), while the administration of MCHR1 antagonist SNAP in the VTA did not influence the index in the SNAP group (75.35 ± 7.46%, *P* > 0.05 vs NS group, *t* = 0.776, *df* = 16). Co-administration of OX-A and MCH decreased the index in the OX-A + MCH group (61.95 ± 9.34%, *P* < 0.001 vs NS group, *t* = 2.528, *df* = 16), which was significantly lower compared to the index of the OX-A group (61.95 ± 9.34%, *P* < 0.001 vs OX-A group, *t* = 5.540, *df* = 16) and slightly higher than that of the MCH group (61.95 ± 9.34%, *P* < 0.05 vs MCH group, *t* = 2.782, *df* = 16). The index of OX-A + SNAP group was higher than the indexes of the NS group (85.92 ± 7.55%, *P* < 0.01 vs NS group, *t* = 3.600, *df* = 16) and the SNAP group (*P* < 0.01 vs SNAP group, *t* = 2.988, *df* = 16), but was not different with that of the OX-A group (*P* > 0.05 vs OX-A group, *t* = 0.367, *df* = 16).

For the HFD mice (Figure 6B), the sucrose preference index of the OX-A group (67.82 ± 8.99%) was higher than the index of NS group (55.99 ± 12.91%, *P* < 0.05, *t* = 2.256, *df* = 16). The administration of MCH in the VTA did not induced a significantly decrease in the index (50.39 ± 6.57%, *P* > 0.05 vs NS group, *t* = 1.160, *df* = 16), but MCHR1 antagonist SNAP obviously increased the index of HFD mice (74.8 ± 6.34%, *P* < 0.01 vs NS group, *t* = 3.922, *df* = 16). The index of OX-A + MCH group was lower than that of the OX-A group (57.40 ± 9.54%, *P* < 0.05 vs OX-A group, *t* = 2.385, *df* = 16), and was not different with those of the NS group (*P* > 0.05 vs NS group, *t* = 0.264, *df* = 16) and the MCH group (*P* > 0.05 vs MCH group, *t* = 1.185, *df* = 16). The mixture of OX-A and SNAP obviously increased the index (80.92 ± 4.20, *P* < 0.001 vs NS group, *t* = 5.509, *df* = 16), which was higher than the indexes of the OX-A group (*P* < 0.01 vs OX-A group, *t* = 3.962, *df* = 16) and the SNAP group (*P* < 0.05 vs SNAP group, *t* = 2.416, *df* = 16).

### Improved hedonic feeding by chemogenetic inhibition of MCH neurons in the HFD mice

**FIGURE 7:**
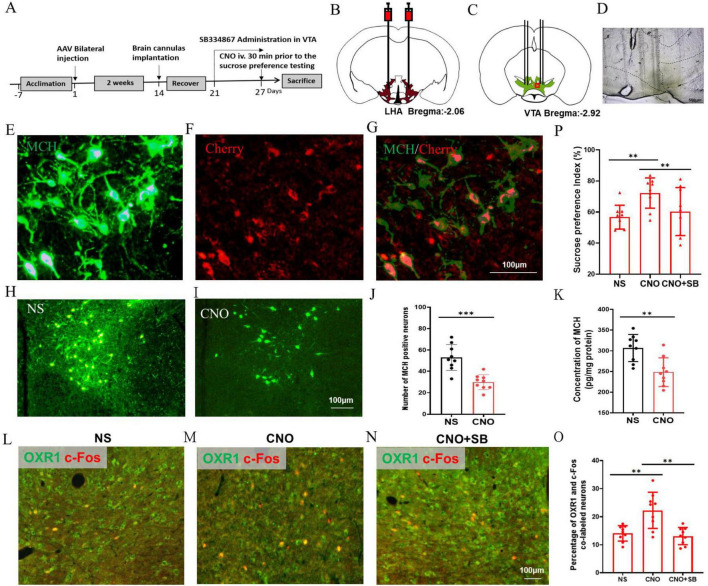
Chemogenetic inhibition of MCH neurons improved the hedonic feeding in the HFD mice. **(A)** The timeline of Experiment 5. **(B)** A schematic diagram of AAV-mMCHp-hM4D(Gi)-mCherry injection into the LHA. **(C)** A schematic diagram of brain cannulae implanted into the LHA. **(D)** A representative image of the brain cannulae trace in the VTA. **(E)** The MCH-immunopositive neurons in the VTA, **(F)** The mMCHp-hM4D (Gi) –mCherry infected neurons in the VTA, **(G)** Co-labeling of mMCHp-hM4D (Gi) -mCherry and MCH in the LHA. **(H)** The MCH-immunopositive neurons in the VTA of NS mice, **(I)** The MCH-immunopositive neurons in the VTA of CNO mice, **(J)** The number of MCH-immunopositive neurons in the VTA, **(K)** The concentration of MCH in the hypothalamus, **(L)** The co-staining of OXR1 and c-Fos in the VTA of NS group, **(M)** The co-staining of OXR1 and c-Fos in the VTA of CNO group, **(N)** The co-staining of OXR1 and c-Fos in the VTA of CNO + SNAP group, **(O)** The percentage of OXR1 and c-Fos co-labeled neurons in the VTA of the NS, CNO and CNO + SNAP mice, **(P)** The sucrose preference index (%) of the NS, CNO and CNO + SNAP mice. Scale bars, 500 μm **(D)**, 100 μm **(E–I,L–N)**. LHA, the lateral hypothalamic area; VTA, the ventral tegmental area; NS, normal saline; CNO, clozapine N-oxide; OXR1, orexin-A receptor 1. SB, SB334867. **P < 0.01, ***P < 0.001, *n* = 9.

To further study the regulation of MCH on the OXR1-immune positive VTA neurons and hedonic behaviors, the chemogenetic technology was implemented to downregulate the expression of MCH in the HFD mice (Figure 7A). AAV2/2-rMCHp-hM4D(Gi)-mCherry-WPRE-pA was bilaterally injected into the LHA (Figure 7B), and the cannulas were also implanted in the LHA for drug administration (Figures 7C, D). 90.34 ± 13.70% of MCH immunopositive neurons were the co-labeled with cherry and 92.83 ± 15.96% virally targeted cells was MCH immunopositive (Figures 7E–G). The number of MCH-expressing neurons in the CNO group was decreased (29.89 ± 7.01) compared to the NS group (53.00 ± 12.17, *P* < 0.001, *t* = 4.938, *df* = 16, Figures 7H–J) and the concentration of MCH in the LHA was also decreased in the CNO group (248.44 ± 34.82 pg/mg protein) compared to the NS group (306.92 ± 32.72 pg/mg protein, *P* < 0.01, *t* = 3.672, *df* = 16, Figure 7K). In the VTA, CNO group had a higher percentage of OXR1 and c-Fos co-labeled cells (22.31 ± 6.44) compared to the NS group (14.12 ± 2.76%, *P* < 0.01, *t* = 3.505, *df* = 16, Figures 7L, M, O), and SB pretreatment decreased the percentage of co-labeled cells to 13.16 ± 3.07% (*P* < 0.01 vs CNO group, *t* = 3.847, *df* = 16, Figures 7M–O). The sucrose preference index was increased by CNO (72.19 ± 9.74) compared to the NS group (56.69 ± 7.68, *P* < 0.01, *t* = 3.751, *df* = 16, Figure 7P), which was inhibited to 60.30 ± 15.47% by OXR1 antagonist SB (*P* < 0.01, *t* = 3.236, *df* = 16, Figure 7P).

## Discussion

According to a report, mice exposed to HFD for 6–9 months displayed heightened anxious-depressive-like behaviors and a significantly decreased sucrose preference compared to normal mice (30). The sucrose preference test, considered the gold standard for assessing hedonic behavior in mice, typically interprets reduced sucrose preference as indicative of “loss of pleasure” (31, 32). In our study, an 8-week HFD led to reduced sucrose preference and diminished c-Fos immunoexpression of VTA neurons toward sucrose when compared to CON mice. C-Fos is an immediate early gene that encodes a transcription factor and is activated by various stimuli. The reduced c-Fos immunoexpression in the VTA suggests decreased neuronal activity in response to sucrose intake among HFD mice. The VTA is a heterogeneous midbrain structure known for its crucial role in reward and motivation processing (33). This implies that the HFD-induced lower activation of VTA neurons might result in “lower pleasure,” potentially leading to reduced interest in palatable food among these mice. However, it is worth noting that HFD mice generally exhibit increased food consumption, highlighting the distinction between “eating behavior” and “hedonic behavior.”

Next, retrograde tracing with FG showed the co-localization of neuropeptides OX-A or MCH in the VTA-projecting neurons within the LHA, a pivotal hypothalamic region responsible for integrating neural signals from both peripheral and central pathways involved in appetite regulation (34). Previous studies have demonstrated that central administration of both OX-A and MCH promotes intake of standard laboratory chow (35–37). In HFD mice, upregulated expression of OX-A and MCH may synergistically contribute to increased “eating behavior.” Regarding reward processing, dose-dependent induction of morphine conditioned place preference (CPP) has been observed with OX-A stimulation (38), while some evidence suggests opposing effects for MCH; for instance, heightened sensitivity to locomotor activating effects induced by DA psychostimulants is exhibited in MCHR1 knockout mice (39). Our findings demonstrate decreased c-Fos expression in VTA-immunopositive neurons expressing OXR1 and MCHR1 in HFD mice, suggesting a potential reduction in “hedonic behavior.”

It has been proved that OX-A enhances the firing rate of DA neurons in VTA (40, 41), while MCH has been shown to directly produce a delayed increase in excitatory input to DA cells *in vitro* (19). In this study, we observed that approximately half of the VTA neurons were activated by OX-A. Furthermore, we assessed the impact of MCH on the OX-E cells and found that it significantly reduced the firing rate of most OX-E cells in the CON mice. These findings suggest that MCH may exert an inhibitory effect on VTA neuron response to OX-A stimulation through coupling with members of the Gi subfamily of G proteins, as previously reported (42). Therefore, based on the higher expression of MCH in the HFD mice, MCHR1 antagonist SNAP-94847 was used to potentiate the effects of OX-A on VTA neurons. The Electrophysiological results showed that MCH might attenuate the effects of OX-A on the VTA OX-E neurons; however, this inhibition was reversed by treatment with SNAP-94847.

Subsequently, animal experiments were conducted to validate the inhibitory effect of MCH on OX-E neurons. In CON mice, the sucrose preference index was significantly lower in the OX-A + MCH group compared to the OX-A group, indicating a reduced hedonic response. The reward system encompasses a complex neural circuit comprising several pertinent nuclei such as the prefrontal cortex, striatum, nucleus accumbens, and VTA. Previous studies have demonstrated that MCH exerts its influence on food, cocaine, and alcohol consumption through different nuclei (43). For instance, MCH promotes chow intake by acting on the shell of nucleus accumbens (44), while localized injections of MCH in the paraventricular nucleus of thalamus enhance alcohol consumption (45). Herein, exogenous administration of MCH resulted in decreased hedonic behavior toward sucrose with or without OX-A co-administration, highlighting its physiological role in modulating reward processing. Furthermore, blockade of overexpressed MCH using SNAP-94847 increased hedonic behavior toward sucrose in HFD mice corroborating with electrophysiological findings and supporting an inhibitory role for MCH within the VTA.

To investigate novel approaches for modulating sucrose preference changes in the HFD mice, we employed chemogenetic manipulation to downregulate the expression of MCH. Our results indicated that reduced MCH levels recovered the response of OXR1 immunopositive neurons in the VTA to OX-A and led to an increased preference for sucrose. The sucrose preference test is commonly used to assess “liking” (46). “Liking” determines which reward is consumed, while “wanting” determines how much of it is consumed (47). Therefore, the increased sucrose preference by inhibiting MCH expression would enhance “liking.” However, the “wanting,” absolute consumption of palatable food, sometimes is different to “liking” (48). Considering food consumption in control and HFD mice, we propose that decreased MCH levels improve VTA neuron activity and promote a greater sense of “liking.” Moreover, due to heightened satiety sensitivity toward sucrose intake, chemogenetically inhibited mice exhibited reduced overall consumption of palatable food consumption, potentially leading to weight loss.

Additionally, OX-A is considered a crucial neuropeptide in stress-related mental disorders, including cognitive changes, disruptions in the sleep-wake cycle, and alterations in appetite (49, 50). For instance, elevated levels of OX-A have been associated with stress-induced binge eating triggered by HFD (51). Another research also showed that activation of LHA-VTA projections due to social stress leads to increased consumption of palatable fats and reducing these projections prevents stress-induced overeating (52). Abnormal elevation of MCH levels caused by chronic unpredictable stress or acute intra-locus coeruleus microinjection induces depression-like behaviors in rats (53). Loss of pleasure is widely recognized as a major characteristic of depressive disorder (54). Therefore, the inhibition of MCH on hedonic feeding induced by OX-A may represent a phenotype resembling depression-like behaviors. In this study, administration of the MCHR1 antagonist SNAP reversed the inhibitory effect exerted by MCH on HFD mice during the sucrose preference test, which aligns with the therapeutic effects observed with MCHR1 antagonists for treating depression (55).

The novel discovery of this study is the inhibitory effect of MCH on OX-E neurons in the VTA. Elevated levels of MCH in HFD mice reduced their preference for sucrose, which was restored by chemogenetic inhibition of MCH neurons. These findings suggest that HFD may modulate hedonic behavior through regulating neuropeptide levels and interactions. Furthermore, targeting antagonists toward MCHR1 could potentially restore sensitivity to hedonic food. Further investigations are necessary to elucidate the impact of MCH alterations on palatable consumption and activation of VTA neurons comprehensively. Moreover, considering the dimorphic nature of the VTA and its association with hedonic behaviors (56, 57), it is crucial to extend this investigation to include female mice. Gaining insights into mechanisms underlying MCH modulation of OX-A-induced hedonic behavior may offer valuable perspectives on reward processing, addictive tendencies, and excessive behaviors. Additionally, a more comprehensive exploration of the interrelationships among neuropeptides should be undertaken, and a cocktail approach involving different agonists or antagonists for neuropeptide receptors may serve as a novel model for drug administration in clinical practice.

## Data Availability

The raw data supporting the conclusions of this article will be made available by the authors, without undue reservation.
